# Development and validation of a modified LibQUAL scale in health sciences libraries: application of Structural Equation Modeling

**DOI:** 10.5195/jmla.2023.1348

**Published:** 2023-10-02

**Authors:** Sirous Panahi, Azam Bazrafshani, Abbas Mirzaie

**Affiliations:** 1 Panahi.s@iums.ac.ir, Associate Professor, Department of Medical Library and Information Science, School of Health Management and Information Sciences, Iran University of Medical Sciences, Tehran, Iran.; 2 Bazrafshani.a@iums.ac.ir, PhD candidate, Department of Medical Library and Information Science, School of Health Management and Information Sciences, Iran University of Medical Sciences, Tehran, Iran.; 3 abmirzaie@gmail.com, PhD candidate, Department of Medical Library and Information Science, School of Health Management and Information Sciences, Iran University of Medical Sciences, Tehran, Iran.

**Keywords:** Consumer services, Quality, Health sciences libraries, LibQUAL, Instrument validity and reliability, Psychometric evaluation, Scale development

## Abstract

**Objective:**

The application of structural equation modeling (SEM), a statistical modeling tool for scale construction and development, is becoming increasingly popular in the health sciences librarianship and information science research. This study explores the application of SEM to health science libraries by describing the development and validation of a modified LibQUAL scale within an Iranian health sciences library setting.

**Methods:**

A literature search was performed across several information sources to identify candidate items to be included in the primary questionnaire. After translation, linguistic validation, and a pilot study, two cross-sectional studies were performed. SEM modeling framework was used for the assessment of the reliability and validity of the modified LibQUAL scale. The internal consistency of the scale was evaluated by measuring Cronbach's alpha coefficient and composite reliability. Exploratory and confirmatory factor analyses were used for the evaluation of the construct validity of the scale. Smart-PLS software was used for statistical modeling.

**Results:**

Composite reliability and Cronbach's alpha coefficient for each scale ranged between 0.90 and 0.95, indicating adequate internal consistency with the LibQUAL scale. Confirmatory factor analysis confirmed the three-factor model of the LibQUAL scale. The convergent validity of the scale was supported, as the average variances extracted for all proposed factors were above 0.50. The discriminant validity was also confirmed using Fornel and Larcker and Heterotrait–Monotrait Ratio (HTMT) methods.

**Conclusion:**

Evaluation of psychometric properties of the translated and locally modified LibQUAL in the Persian language indicated adequate reliability, factorial validity, and stability of this instrument for Iranian health sciences libraries.

## INTRODUCTION

LibQUAL is a well-recognized instrument for measuring the quality of services in academic libraries [1, 2]. LibQUAL was developed by the collaboration of Texas' academic libraries and the Association of Research Libraries (ARL) to help librarians understand the expectations and needs of library users; assess the quality of library services from their perspectives; and thus improve the quality of services and better meet library users' information needs [3]. The present version of the LibQUAL survey consists of twenty-two core questions characterized under three important areas or dimensions including the Affect of Service (AS), Information Control (IC), and Library as Place (LP).

Since its inception in 2000, about 1.5 million library users from 1,200 academic institutions across the world have participated in LibQUAL surveys to evaluate the quality of their library services. To date, translation and adaptation of the English language questionnaire into a variety of languages including Spanish, French, Welsh, Swedish [4], Arabic, and Urdu [5] have provided librarians with the opportunity to compare assessment results from other academic institutions and recognize best practices in library services across the world. However, the credibility of results from these assessments depends on the reliability and validity of the research instruments used for data collection.

Research on the reliability and validity of LibQUAL assessments by ARL and Texas A&M has been well documented in the literature [1, 2, 6, 7], yet limited reports are published by libraries on cross-cultural implementation of locally modified versions and their reliability and validity assessments. Many librarians use local surveys that fail to produce accurate and reliable data [6]. Since the accuracy and validity of the data obtained from LibQUAL are directly related to its psychometric properties in a new context, it is necessary to examine its validity and reliability through rigorous methodological studies before using a translated version of this tool. This helps ensure the accuracy of the tool in assessing service quality while considering the impact of linguistic, cultural, and ethnic factors. Iranian academic libraries have used LibQUAL surveys extensively to assess quality of services over the years [8-11]. However, existing evidence about the reliability and validity of this instrument in the Persian language is limited to some preliminary and descriptive studies.

Assessing the validity of a translated research instrument such as LibQUAL requires the use of advanced quantitative analytic tools such as structural equation modeling (SEM). SEM allows for the testing of complicated, multifaceted theories, and constructs. Further, it also enables the assessment of relationships between observed variables and underlying theoretical constructs (i.e., latent variables) [12]. SEM can be used to confirm the factor structure of a newly developed instrument or the use of an existing instrument with a new population [13].

The present study intends to familiarize health science librarians with the application of SEM, as well as provide an applied approach to conducting SEM analysis. In particular, this study describes a statistical foundation for developing and validating a research instrument in the context of health science libraries (LibQUAL scale). The stages comprise item generation and identification, instrument construction, and SEM modeling. The study proceeds as follows: we describe the methodology and procedure for instrument development and assessment; we present in detail the development process, the analysis through SEM framework and, the result of the developed instrument. Subsequently, we highlight our contribution to research and practice and the entailed limitations. Finally, we conclude the study with suggestions for further development.

## METHODS

**Figure 1:**
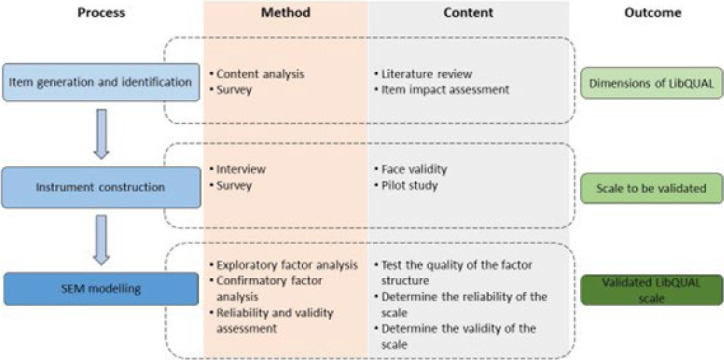
Development process of the LibQUAL scale using structural equation modeling (SEM) framework

Figure 1 illustrates the preliminary steps that were utilized before the application of SEM modeling (steps 1-4). Following development of a scale and the questionnaire, the face validity of the developed scale was investigated. A pilot study then was executed to identify the indicator variables and appropriate samples. After the execution of all preliminary steps, the adequate framework was prepared to be applied in the further statistical analysis and conduction of SEM modeling.

### Stage 1: Item generation and identification

#### Literature search and review

The first step in instrument development is to determine what the tool will measure by identifying and defining the components of interest. Academic librarians often glean information about the quality of library services by observation and by asking library users. For example, a librarian may ask the users about their satisfaction with the library services or the performance of librarians. Therefore, an instrument designed to determine high quality services could measure the concepts of satisfaction, librarian performance, and the library. Alternatively, existing instruments driven from theories could be used for evaluating quality of library services. In addition, a comprehensive review of the literature to determine what is known about concepts should be conducted.

To identify candidate items reflecting service quality in academic libraries, a literature search was conducted to identify relevant research publications and project reports about LibQUAL. The literature search was conducted using several information sources: Library and Information Science Abstracts (LISA), Emarald Insight, Google, Google Scholar, as well as hand-searching of relevant literature in Persian language. Keywords included “LibQUAL,” “library service assessment,” and “library service quality.”

Reports of LibQUAL survey from any library published in English or the Persian languages from January 2000 to December 2018 were retrieved and organized in an EndNote library. The retrieved records were then screened to identify different versions of LibQUAL surveys. Distinct or unique translations and adaptations of LibQUAL surveys were further reviewed, and relevant items were extracted and inserted in an excel file. Then, extracted items were reviewed, duplicate items were removed and distinct items were selected for translation. Out of 326 retrieved unique publications, 13 publications were selected for final review. Subsequently, a total of 50 questions (22 questions from the original LibQUAL survey combined with 28 questions from the local library surveys in English or Persian) were identified through extensive searching of relevant databases.

The standard procedure for translation included forward-backward translation, carried out independently by native-speaker of target language. In case of any disagreement or difference in forward-backward translation, it was attempted to ask a third expert to resolve disagreement. Existing translations of LibQUAL surveys in Persian language were also included and compared with the original English versions to identify the best translation. Then the translated items were reviewed by a group of experienced librarians, scale developers and professional translators from Iran University of Medical Sciences. In this step, the authors tried to achieve conceptual, semantic, and normative equivalence instead of word-by-word translation.

#### Item impact assessment

When the translation was completed, a survey was conducted in January-February 2019 to explore perceptions of library users about the importance of candidate items retrieved from literature. Library users were recruited from Iran University of Medical Sciences with a population of more than 750 faculty members, 6,000 students and 70 librarians. Participation in this survey was voluntary and verbal consent was obtained before completion and returning the questionnaire. A convenience sample of 36 students, 25 faculty members and 5 librarians were recruited from two college libraries. Participants were required to rate the importance of any candidate item on a 1-5 Likert scale where 1 indicated extremely not important and 5 extremely important for service quality. Then, an item-impact score was measured using this formula: percentage of participants who ranked the item as 4 or 5☐ average of importance score for the item. An impact score above 1.5 was considered as appropriate. After eliminating 15 items from the process of item-impact score assessment, the translated version of the LibQUAL incorporated 35 items.

### Stage 2: Instrument construction

As a result of identifying and defining the characteristics of the library service quality, an initial pool of 35 items was developed as a conceptual framework or blueprint for the instrument. The framework reflected the three major categories or domain of LibQUAL survey. These major domains involved “Affect of service” (AS), “Information control” (IC) and “Library as place” (LP).

#### Face validity

Five experts (three librarians, one linguist and one epidemiologist) were consulted to highlight whether there were any errors in the categorization and should any candidate items need change in categorization. Therefore, the final translated version of the LibQUAL scale consisted of 35 core items in three dimensions: AS, IC and LP.

#### Pilot study

A pilot study with a sample of 30 library users was conducted.

### Stage 3: SEM modelling

The next step in the scale development process is validation. Validity is defined as the degree to which the items measure the concept of interest. Construct validity establishes support for the measures ability to function for its intended purpose. Factor analysis is a method to determine construct validity. Quality of library services cannot be measured directly but is measured using a variety of components like “Affect of Service,” “Information Control,” and “Library as Place.” The components or factors aid in describing and understanding more complex phenomena like quality. Factor analysis is a statistical process that reduces data from a large set of interrelated components or factors into a smaller number of subcomponents that describe the relationships among variables.

There are two types of factor analysis: exploratory factor analysis (EFA), and confirmatory factor analysis (CFA). EFA is usually used as a preliminary step to analyze the nature of latent variables and to provide a preliminary understanding of the relationships between the measured variables and the corresponding latent constructs. EFA is useful when we are only interested in searching for a structure among a set of variables or when we want to employ the data reduction method (reducing the number of items in a questionnaire). However, EFA is not served to test a certain theory. CFA is usually used to inspect how well the indicator variables represent the latent factors [12]. CFA is useful, when we already have some preconceived ideas about the actual structure of the data, based on certain theoretical support or prior research. In this regard, the confirmatory approach can assess the degree, which explains how the data meet (fit) the predefined structure. Dragan and Topolšek note that “the main difference between the EFA and CFA is reflected in the fact that in the case of CFA, the factor structures are hypothesized in advance and verified empirically, while in the case of EFA, these structures are derived from the data” [13].

#### Exploratory factor analysis (EFA)

As noted in Figure 1, EFA was employed as the first step for analyzing the latent constructs and to provide a preliminary insight into the relationships between the measured variables and the corresponding latent factors. To do this, faculty members and students (n=155) from Iran University of Medical Sciences were surveyed in April 2018 (Study A). The questionnaire consisted of 4 demographic questions and 35 statements drawn from the initial version of the LibQUAL. Demographic questions included age, gender, field of study, and frequency of library services utilization. The pilot version of LibQUAL included 35 items and 3 constructs: “Affect of service (AS)” (12 items), “Information Control (IC)” (13 items), and “Library as a Place (LP)” (10 items). Respondents were asked to rate library services and the library's perceived service performance on a scale of 1-9. Questionnaires were delivered and collected by three students trained as research assistants before data collection. Respondents completed questionnaires individually at their convenience, and assistants explained any unclear questions without inducing the respondents' answers.

#### Confirmatory factor analysis (CFA)

CFA was conducted to confirm the LibQUAL factor structures based on the EFA investigation (Study A). Study B was conducted in November 2019, with another group (N=200) of faculty members and students from Shahid Beheshti University of Medical Sciences as well as Tehran University of Medical Sciences. Of the 200 library users invited to the survey, 197 users (response rate= 98.5%) completed and returned the questionnaire to the interviewers.

This study adapted the SEM modelling technique to explore the relationship between theoretical constructs and measured variables [14, 15]. SEM modelling involves a two-stage process. In the first stage, the measurement model is constructed and validated by the means of CFA (14), while in the second stage the design of the whole structural model is completed by adding the structural part of model and appropriately validating the entire model structure [15].

#### Reliability and validity assessment

CFA analysis involves investigating convergent validity discriminant validity, as well as the reliability. Smart PLS 3.0 [16] was used to assess internal consistency, convergent and discriminant validity. Table 1 summarizes the thresholds for the measures of convergent and discriminant validity and reliability in the CFA analysis.

**Table 1:** Thresholds for measures of validity and reliability in the CFA analysis

Indicator	Acceptance level	Description
**Reliability**		
Internal consistency	Composite reliability (CR) > 0.7 Cronbach's alpha > 0.7	Higher values indicate good reliability (contingent upon number of items)[17]
Average Variance Extracted (AVE)	0.5 and above	AVE above 0.50 confirms the convergent validity and indicates items explain more variance in the constructs[18].
**Construct validity**		
Convergent Validity	Standardized factor loading >0.5 or preferably 0.7, AVE > 0.5, CR > 0.7	Higher values indicate that the items studied reflect the constructs that they are intended to measure[19, 20]
Discriminant validity	AVEs > squared inter-construct correlation (SIC) Or square root of AVE > inter-construct correlation	Higher values indicate that a given construct is sufficiently different from the other constructs[20]

#### Reliability

Reliability measures the stability of an instrument. An instrument is considered reliable if results are consistent regardless of testing circumstances. Reliability analysis is performed after the instrument has been administered to a test group to examine internal consistency. Internal consistency determines the correlation of individual test items with each other, in which a perfect relationship among items would be 1.0. Internal consistency reliability assumes that an instrument is designed to measure a particular characteristic and test items devised to measure the characteristic should also relate to each other. Composite Reliability (CR) and Cronbach's alpha coefficient are two preferred indices for internal consistency. CR values need > 0.7 to ensure adequate or sufficient internal consistency (Table 2). Cronbach's Alpha α> 0.7 is another measure of the reliability of items measuring a construct. A large alpha suggests high internal consistency, meaning that the test is measuring one attribute. A small alpha suggests item correlations are low and performance on one item is not predictive of performance on other items (Table 2). Table 3 describes the values of Cronbach's Alpha and CR for major constructs of the proposed LibQUAL scale in this study.

**Table 2:** Demographic information of the library users participated in the reliability and validity assessment of the proposed LibQUAL scale in Persian

Indicators	Study A, (n=134) n (%)	Study B, (n=197) n (%)
Age		
18-22	39 (29.1)	108 (54.8)
23-30	50 (37.3)	63 (32.0)
31-44	38 (28.3)	20 (10.2)
45-65	7 (5.2)	6 (3.0)
Gender		
Male	47 (35.1)	66 (33.5)
Female	87 (64.9)	131 (66.5)
Sample group		
Students	112 (83.6)	182 (92.4)
Undergraduate	40 (29.9)	57 (28.9)
Graduated	72 (53.7)	125 (63.5)
Faculty members	22 (16.4)	15 (7.6)
Discipline		
Medicine	30 (22.3)	2 (1.1)
Dentistry	1 (0.7)	63 (32.0)
Pharmacy	2 (1.5)	78 (39.6)
Nursing & Midwifery	13 (9.7)	6 (3.0)
Rehabilitation Science	5 (3.7)	-
Health Informatics & Management	23 (17.2)	16 (8.1)
	20 (14.9)	12 (6.0)
Public Health	40 (29.8)	20 (10.2)
Paramedical sciences		

**Table 3:** Reliability and convergent Validity of the proposed LibQUAL scale

Construct	Item code	Outer loadings	AVE	Composite Reliability	Cronbach's Alpha
Affect of service	AS1	0.70	0.72	0.96	0.95
	AS2	0.84			
	AS3	0.86			
	AS4	0.89			
	AS5	0.83			
	AS6	0.85			
	AS7	0.89			
	AS8	0.88			
	AS9	0.89			
Information Control	IC1	0.78	0.63	0.92	0.90
	IC2	0.74			
	IC3	0.77			
	IC4	0.83			
	IC5	0.79			
	IC6	0.82			
	IC7	0.83			
Library as Place	LP1	0.83	0.69	0.94	0.92
	LP2	0.84			
	LP3	0.86			
	LP4	0.84			
	LP5	0.89			
	LP6	0.82			
	LP7	0.70			

#### Convergent validity

Convergent validity measures how closely the new scale is related to other variables and other measures of the same construct [21]. Convergent validity can be evaluated by three tests including 1) standardized factor loadings; 2) Average Variance Extracted (AVE); and 3) Composite Reliability (CR). Composite reliability is established when it exceed the recommended threshold of 0.70 [22].

Table 4 describes the findings of Outer Loading, CR and AVE values. As mentioned before, in this study several items were eliminated based on the AVE value requirement for each construct that should exceed 0.7 [21].

**Table 4:** Convergent, and discriminant validity and reliability assessment of the final the LibQUAL scale in Persian

Construct	Fornell-Larcker Criterion			Heterotrait–Monotrait Ratio (HTMT)		
	Affect of Service	Information Control	Library as Place	Affect of Service	Information Control	Library as Place
Affect of service	0.85					
Information Control	0.43	0.80		0.46		
Library as Place	0.53	0.70	0.83	0.57	0.76	
Service quality	0.70	0.75	0.79	0.72	0.78	0.82

#### Discriminant validity

The discriminant validity measures how well the constructs tested differed from the other constructs. This analysis can determine how much one construct correlates with another construct and how many items can represent a single construct [21]. This study used three tests to measure discriminant validity including 1) factor Loading; 2) Fornell & Larcker Criterion; 3) Heterotrait-Monotrait Ratio (HTMT).

Factor loading is defined as the correlation coefficient for the items and constructs. Factor loading shows the variance explained by the variable on that particular factor. In the SEM approach, loading value of the construct should be greater than all the AVE values [23]. Fornell-Larcker Criterion is one of the most popular techniques used to test the discriminant validity. According to this criterion, the square root of the construct's AVE must be greater than the correlation between the construct and any other construct [21]. Heterotite-Monotrait Criteria Analysis (HTMT) should not be exceeded the 0.85 [24] or 0.9 [25] minimum threshold to indicate satisfactory discriminant validity.

#### The Structural model

The next step in SEM modelling is to investigate the structural model. For this purpose, the corresponding measurement model was converted to the structural model at first. In the next step, the structural model validity was assessed, where the goodness of fit indices and the direction of all paths were investigated, as well as the structural parameters were estimated.

In this study, R Square and path coefficient measures were used to examine the relationships among the constructs (AS, IC and LP) and LibQUAL scale. R square explains the variance in a construct explained by its included items. The minimum threshold for R square value is 0.1*, indicating that the variance explained of a particular construct to be deemed adequate. When the structural model (path model) of the LibQUAL scale was developed, the next step was to evaluate its quality of fitting the observed data. In this regard, the developed structural model of the LibQUAL scale was assessed using the goodness-of-fit measures. Standardized Root Mean Square Residual (SRMR) is a measure that examines the goodness of measurement models. SRMR values less than 0.10 or 0.08 are considered as a good fit [22].

## RESULTS

134 library users participated in this study and completed the initial version of the LibQUAL scale (a response rate of 86 percent). Table 2 describes the demographic information of respondents who participated in Study A and Study B accordingly.

In Study A, major components or factors of the translated LibQUAL scale with 35 items were evaluated (Appendix 1, Table B). Factor loading indicates the amount of variance of each item in the construct model.

Lower loading of item indicates that such item may not be an effective measure of its construct and should be dropped from the model. According to this analysis, three indicators including AS1 (0.597), AS2 (0.661), and LP9 (0.587) were eliminated from the questionnaire (Appendix A, Table C). In order to collect further information about the convergent and discriminant validity and the dimensionality of the LibQUAL scale, we conducted a subsequent study with more respondents to increase the sample size.

Data from the Study B were analysed and the factor loading of the remaining 32 items were subsequently calculated. The results of this analyses indicated that the factor loadings of nine items were lower than 0.7 (Appendix A, Tables D and E). Therefore, these items were eliminated from the analysis. Removal of these items led to a three-factor solution with a total of 23 items.

Appendix A, Table A describes the process of eliminating candidate items across different stages in this study. Appendix B provides some explanations about the statistical terms used in this study.

Cronbach's alpha coefficient and composite reliability of all composite constructs were greater than the acceptable level of 0.70, demonstrating appropriate internal consistency of the proposed LibQUAL scale in Persian (see Table 3). Confirmatory indicator loading of 23 Items of the proposed LibQUAL scale in Persian was significant with a range of 0.70 – 0.89. In addition, the AVE of the three constructs of LibQUAL scale was higher than 0.50 and confirmed the convergent validity of the model. Convergent validity can also be assessed by estimating the composite reliability of the LibQUAL constructs. The composite reliability for the LibQUAL constructs exceeds the recommended level of 0.70 ranging from 0.90 – 0.95 (see Table 3).

The cross loading of the respective factors (AS, IC and LP) in Table 4 are greater than those factors' AVE (reported in table 3). For example, the factor loading of “AS” is 0.85 which is greater than its respective AVE of 0.72. Since the factor loading is greater for all the factors than the AVE of the respective construct, discriminant validity is confirmed. Table 4 also describes the higher AVE squared values compared to the correlation values for each other construct after some items that did not meet the factor loading conditions were eliminated from the proposed LibQUAL scale. The Fornell-Larcker value for ”AS” is 0.85, which is higher than all other calculated values for other constructs. Finally, the HTMT value was lower than the acceptable level of 0.90, indicating that discriminant validity was established among all reflective constructs (see Table 4).

For example, as shown in Table 5, the estimated R square for Library as Place was 0.63. This would mean that 63% change in Library as Place can be explained by its included items (LP1-LP7).

**Table 5:** Assessment of structural model of the LibQUAL scale in Persian

Variables	R square	Path coefficient	T value	Significant level
Affect of service	0.49	0.701	19.58	0.001
Information control	0.56	0.747	24.04	0.001
Library as place	0.63	0.792	23.75	0.001

A path coefficient indicates the direct effect of a construct (AS, IC, LP) on another variable (service quality). For example, according to table 6 (see supplementary materials), Library as Place has the largest, positive effect on the overall serve quality. The estimated R Square and path coefficients were higher than the acceptable level[26], demonstrating the robustness (performance with high quality) of the structural model. The t-values retrieved from bootstrapping (using random samples from the original data) in Smart-PLS software indicated significance associations between the Constructs (AP, IC and LP), and the overall service quality score (P<0.01). Appendix A-Figure A illustrates the final structural model of the proposed LibQUAL scale in Persian.

According to our findings, the SRMR measure for the LibQUAL scale was lower than the recommended threshold (SRMR=0.06), demonstrating adequate fitness of the LibQUAL model in Persian. Normed Fit Index (NFI) has been also recommended for estimating the goodness-of-fit of the measurement models. The NFI usually ranges between 0 and 1. The closer the NFI to 1, the better the fit [27]. The NFI measure estimated for the LibQUAL scale also confirmed the acceptable goodness of the model (NFI=0.85). Based on results of the goodness-of-fit measures of the LibQUAL scale, the measurement model demonstrates adequate fitness for providing a platform for the development and assessment of the LibQUAL structural model.

## DISCUSSION

The current study aspired to develop and locally modify the LibQUAL scale in Persian and assess its statistical reliability and validity when used to assess quality of services among Iranian library users. Face and content validity of the translated and locally modified LibQUAL scale in Persian language were approved with some modifications. Internal consistency of the LibQUAL scale was approved by composite reliability and Alpha Cronbach's coefficient. Construct validity of the scale was confirmed by CFA using structured equation modelling.

Our findings confirmed the three-factor construct of the final LibQUAL in Persian language: AS, IC and LP. The three-factor construct of the LibQUAL scale was frequently reported in a volume of research reported by for different languages including the French [28] and Urdu [5]. These findings are also consistent with research findings produced by Thompson, Cook and Kyrillidou (2005) and Thompson, Kyrillidou and Cook (2008). There is a piece of evidence suggesting a four-factor construct for LibQUAL in Spanish language [6]. However, the three-factor construct of the LibQUAL scale reported by a range of recent investigations seemed more robust and parsimonious.

The results of reliability assessment indicated that all three factors of the LibQUAL scale in Persian language had high composite reliability and Alpha Cronbach's coefficient in Iranian Context. According to our findings, Alpha Cronbach's coefficients for AS, IC and LP were greater than similar studies in Asia [5] and other countries [29-33]. Therefore, our results of the internal consistency and reliability of the LibQUAL scale are highly compatible with available research findings from other languages and support the idea that the translated and locally modified LibQUAL is highly reliable in Persian language.

Our findings are based on data collected from cross-sectional survey of library users from particular institutional and geographical contexts that could affect the generalizability of the results. However, the statistical procedures and methods used in this study could be generalizable to other studies seeking to to develop research instruments or evaluate theoretical models in social science contexts. The SEM approach has several applications in validating theoretical models, reliability assessments, and analyzing complex models using empirical data. However, the complexity of using SEM techniques comes with statistical and interpretational challenges and problems. Therefore, we hope that this study may serve as a foundation for health science librarians and medical library and information experts who may be interested in developing and validating scales for library service assessments.

## CONCLUSION AND IMPLICATIONS

This study aimed to assess the reliability and validity of the LibQUAL scale in Persian language using structured equation modelling. Based on the findings of this study, the translated and modified version of the LibQUAL in Persian is a validated and reliable tool. It can be used to measure quality of services in the Iranian academic libraries and in a variety of disciplines such as health sciences. The face and content validity of the scale is confirmed by some modifications. Convergent validity, reliability, and discriminant validity of the 23 items of the translated and modified LibQUAL scale was approved by CFA using partial least square method.

Health science librarians in Iran and most parts of the world are facing significant challenges in meeting the emerging demands of healthcare organizations and users. Health and hospital libraries provide a unique opportunity for health science librarians to develop professional roles and responsibilities that support clinical research and practice. It's important for librarians to understand what services their clients value, and how well their clients perceive the library to be performing those services. Given the importance of those two things, a survey like LibQUAL can be very valuable for librarians. LibQUAL can serve as a good foundational survey for health sciences librarians across the globe, but only if it is grounded in the native language and reflective of the local culture in which it's being implemented. In this context, the statistical procedures and results described in this study may enable health science librarians in other countries to develop and validate their own locally modified surveys to recognize the relationships between service dimensions, to weigh their importance, and to assess their impact on their users' satisfaction.

## Data Availability

The datasets used and/or analysed during the current study are freely available from a GitHub repository, available from https://github.com/bazrafshan/LibQUAL.git.

## SUPPLEMENTAL FILES

**Appendix A:** Tables and Figures

**Appendix B:** Key Statistical Terms
