# Supplementary material for: Development and validation of a modified LibQUAL scale in health sciences libraries: application of Structural Equation Modeling
Source: J Med Libr Assoc. 2023 Oct 2;111(4):792–801. doi: 10.5195/jmla.2023.1348 (PMC10621718; doi:10.5195/jmla.2023.1348)

Appendix A: Tables and Figures

Table A Item elimination across different stages of development, reliability and validity assessment of the proposed LibQUAL scale in Persian

CODE	ITEM	ITEMS SELECTED FROM IMPACT ASSESSMENT	ITEMS SELECTED FROM STUDY A (EFA)	ITEMS SELECTED FROM STUDY B (CFA)	ITEMS IN THE FINAL LIBQUAL SCALE
**AS1-1**	Topical advice should be provided by a subject specialist librarian	*			
**AS2-1**	It is possible to provide feedback regarding the quality of library services and staff performance	*			
**AS3-1**	The number of library personnel should be appropriate				
**AS4-1**	Employees who create a sense of trust and confidence in users	*	*	*	*
**AS5-1**	Librarians pay attention to the needs of individual visitors	*	*	*	*
**AS6-1**	Employees who deal with users with respect and politeness	*	*	*	*
**AS7-1**	Librarians are always ready to respond to the questions they are asking	*	*	*	*
**AS8-1**	Employees who have sufficient knowledge to answer the questions of the respondents	*	*	*	*
**AS9-1**	Employees who treat and respond to clients in a caring fashion	*	*	*	*
**AS10-1**	Employees who understand the needs of their clients	*	*	*	*
**AS11-1**	Librarians who are always willing to help the visitors	*	*	*	*
**AS12-1**	Librarians who have high dependability in solving the service problems of the users	*	*	*	*
**AS13-1**	Librarians should be able to provide facilities for disabled people				
**AS14-1**	Librarians should be able to provide research advice to users if needed				
**AS15-1**	The library should use an efficient information retrieval system	*	*		
**AS16-1**	It is possible to hold educational workshops in the library				
**IC1-1**	The lending system of resources should be accurate.	*	*		
**IC2-1**	Cataloging of resources should be done carefully so that the error of retrieving unrelated resources in the library retrieval system is minimized.	*	*		
**IC3-1**	It is possible to access audiovisual resources in the library	*	*		
**IC4-1**	New resources are regularly added to the library collection	*	*		
**IC5-1**	There should be a reasonable balance between library resources in different subjects				

**IC6-1**	It is possible to access the abstracts of theses and dissertations	*	*		
**IC7-1**	The existence of facilities that provide access to electronic information sources from home or work	*	*		
**IC8-1**	The existence of a library website that helps me locate the information I need	*	*	*	*
**IC9-1**	Are printed resources that are necessary for my work	*	*	*	*
**IC10-1**	I have the electronic resources I need	*	*	*	*
**IC11-1**	The availability of modern equipment that allows me to easily access my information	*	*	*	*
**IC12-1**	Having easy-to-use access tools that allow me to find information personally	*	*	*	*
**IC13-1**	The availability of facilities that makes my information easy to access	*	*	*	*
**IC14-1**	Available printed or electronic publications that are necessary for my work	*	*	*	*
**LP1-1**	There is a space that encourages learning and innovation	*	*	*	*
**LP2-1**	A space that is quiet and suitable for individual activities	*	*	*	*
**LP3-1**	Library space is comfortable, pleasant and attractive	*	*	*	*
**LP4-1**	The library space is a suitable place for collective communication, group learning, and group study				
**LP5-1**	It is possible to have drinks and food in the library				
**LP6-1**	The library space is enough	*	*	*	*
**LP7-1**	Physical library facilities such as computers, desks and chairs, and lighting suitable for study	*	*	*	*
**LP8-1**	Moving in the library environment is easy	*	*	*	*
**LP9-1**	The library space should be clean				
**LP10-1**	The temperature of the library environment should be suitable				
**LP11-1**	Access to directories and library service instructions is provided	*	*	*	*
**LP12-1**	Access to wireless internet (WiFi) in the library space	*	*		
**LP13-1**	It is possible to provide services to disabled people in the library	*	*		
**LP14-1**	The ventilation of the library should be suitable				
**LP15-1**	A resource drop box should be available, especially for non-office hours				
**LP16-1**	Each user has a dedicated closet				
**LP17-1**	There is a photocopier in the library				
**LP18-1**	The hardware and software facilities of the library should be suitable	*			
**LP19-1**	It is possible to access library resources remotely				

Table B Descriptive statistics of the primary 35items of the proposed LibQUAL scale inPersian

Item code	Min	Max	Mean	Std. Deviation
AS1	1	9	4.83	2.00
AS2	1	9	5.14	2.13
AS3	1	9	5.39	2.19
AS4	1	9	5.29	2.28
AS5	1	9	5.81	2.29
AS6	1	9	5.64	2.12
AS7	1	9	5.51	2.13
AS8	1	9	5.50	2.12
AS9	1	9	5.73	2.04
AS10	1	9	5.83	2.09
AS11	1	9	5.76	2.09
AS12	1	9	5.86	2.11
IC1	1	9	5.96	2.04
IC2	1	9	5.92	2.08
IC3	1	9	5.34	2.22
IC4	1	9	5.71	2.05
IC5	1	9	5.98	1.95
IC6	1	9	5.70	2.08
IC7	1	9	6.15	1.95
IC8	1	9	5.98	2.01
IC9	1	9	5.97	2.01
IC10	1	9	5.77	2.18
IC11	1	9	5.92	1.97
IC12	1	9	5.76	1.98
IC13	1	9	5.94	2.08
LP1	1	9	5.99	2.04
LP2	1	9	5.97	2.35
LP3	1	9	6.21	2.22
LP4	1	9	6.13	2.30
LP5	1	9	6.40	2.25
LP6	1	9	6.56	1.98
LP7	1	9	6.29	2.11
LP8	1	9	6.52	2.19
LP9	1	9	5.23	2.28
LP10	1	9	6.37	2.10

Table C Factor loading, reliability and validity analysis of 35 Items of the proposed LibQUAL scale in Persian

Construct	Item code	Factor loadings	AVE	Cronbach's alpha	Composite reliability
Affect of Service	AS1	0.597	0.657	0.951	0.958
	AS2	0.661			
	AS3	0.821			
	AS4	0.852			
	AS5	0.853			
	AS6	0.828			
	AS7	0.878			
	AS8	0.854			
	AS9	0.801			
	AS10	0.865			
	AS11	0.829			
	AS12	0.840			
Information Control	IC1	0.763	0.618	0.948	0.955
	IC2	0.768			
	IC3	0.777			
	IC4	0.794			
	IC5	0.733			
	IC6	0.720			
	IC7	0.790			
	IC8	0.806			
	IC9	0.806			
	IC10	0.829			
	IC11	0.854			
	IC12	0.850			
	IC13	0.716			
Library as Place	LP1	0.859	0.672	0.944	0.953
	LP2	0.845			
	LP3	0.871			
	LP4	0.883			
	LP5	0.907			
	LP6	0.843			
	LP7	0.830			
	LP8	0.811			
	LP9	0.587			
	LP10	0.709			

- Highlighted items were removed from the questionnaire due to loadings smaller than the acceptable level of 0.70.

Table D Descriptive Statistics of Primary 32 Items of proposed LibQUAL scalein Persian

Item code	Minimum	maximum	Mean	Std. Deviation
AS1	1	9	0.6124	0.3778
AS2	1	9	0.7599	0.4296
AS3	1	9	0.7943	0.4069
AS4	1	9	0.7069	0.4089
AS5	1	9	0.6777	0.3866
AS6	1	9	0.7766	0.4484
AS7	1	9	0.7243	0.4577
AS8	1	9	0.7793	0.3632
AS9	1	9	0.8371	0.3260
AS10	1	9	0.5765	0.3008
IC1	1	9	0.2985	0.2743
IC2	1	9	0.2140	0.2036
IC3	1	9	0.9081	0.1834
IC4	1	9	0.8596	0.2274
IC5	1	9	0.8259	0.1950
IC6	1	9	0.8737	0.2039
IC7	1	9	0.8630	0.1854
IC8	1	9	0.8419	0.1979
IC9	1	9	0.8703	0.1548
IC10	1	9	0.8800	0.1526
IC11	1	9	0.8439	0.1506
IC12	1	9	0.8879	0.1386
IC13	1	9	0.8559	0.1640
LP1	1	9	0.8158	0.1932
LP2	1	9	0.8096	0.2299
LP3	1	9	0.5644	0.3907
LP4	1	9	0.6541	0.3799
LP5	1	9	0.6016	0.4081
LP6	1	9	0.5977	0.4106
LP7	1	9	0.5243	0.3666
LP8	1	9	0.5213	0.3922
LP9	1	9	0.4262	0.3112

Table E Factor loading, reliability and validity analysis of 32 Items of the proposed LibQUAL scale in Persian

Construct	Item code	Factor loadings	AVE	Cronbach's alpha	Composite reliability
**Affect of Service**	AS1	0.7717	0.643	0.972	0.918
	AS2	0.9293			
	AS3	0.9486			
	AS4	0.8727			
	AS5	0.8253			
	AS6	0.9645			
	AS7	0.9091			
	AS8	0.9009			
	AS9	0.8955			
	AS10	0.6742			
**Information Control**	IC1	0.3619	0.665	0.973	0.925
	IC2	0.2633			
	IC3	0.6381			
	IC4	0.6004			
	IC5	0.6528			
	IC6	0.6954			
	IC7	0.8771			
	IC8	0.8667			
	IC9	0.8895			
	IC10	0.9025			
	IC11	0.8603			
	IC12	0.9048			
	IC13	0.8811			
**Library as place**	LP1	0.7704	0.625	0.942	0.948
	LP2	0.7700			
	LP3	0.7747			
	LP4	0.8668			
	LP5	0.8351			
	LP6	0.8348			
	LP7	0.7014			
	LP8	0.5277			
	LP9	0.5630			

Figure A The structural model of the LibQUAL scale in Persian: Items of scale; AS: Affect of service; IC: Information control*;* and LP: Library as place

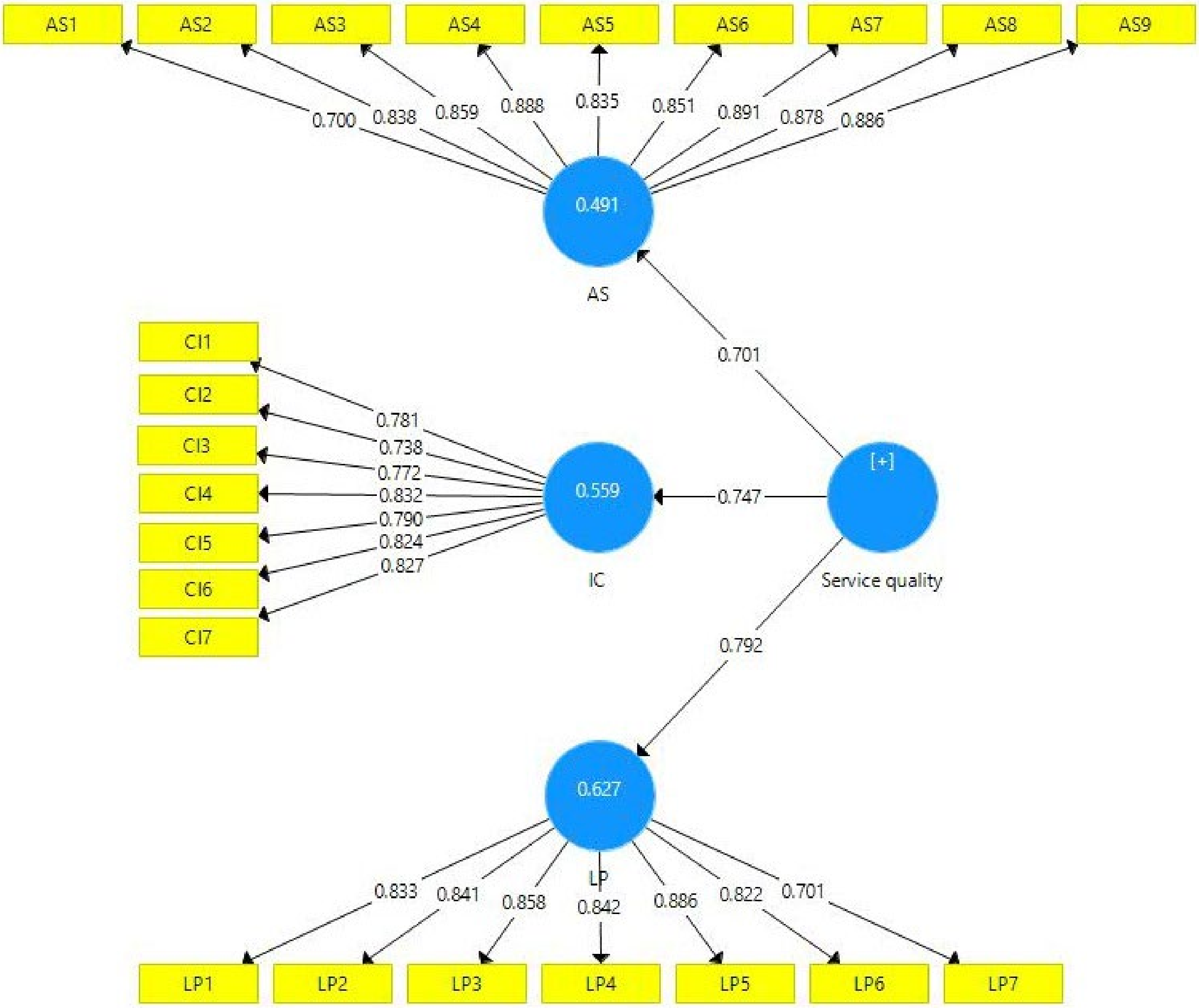

Supplement: Supplementary file 1 — Appendix A: Tables and Figures [file jmla-111-4-792-s01.pdf]
