# Supplementary material for: Development and validation of a modified LibQUAL scale in health sciences libraries: application of Structural Equation Modeling
Source: J Med Libr Assoc. 2023 Oct 2;111(4):792–801. doi: 10.5195/jmla.2023.1348 (PMC10621718; doi:10.5195/jmla.2023.1348)
Supplement: Supplementary file 2 — Appendix B: Key Statistical Terms [file jmla-111-4-792-s02.pdf]

Appendix B: Key Statistical Terms

Construct validity

Construct validity is used to evaluate the validity of a test, questionnaire, or instrument. It is often used in the social sciences, education, and psychology. Construct validity refers to how well a test (i.e., A questionnaire) measures what it is supposed to measure. In other words, is the test constructed in a way that it successfully tests what it claims to test? For example, we might try to find out whether our library services are meeting the users' expectations and needs. Construct validity would measure whether your LibQUAL scores are measuring the quality of library services.

There are two types of construct validity: convergent and discriminant validity.

The **convergent validity** confirms whether the scores of a construct (i.e., affect of service) "make sense" in relation to the scores of other similar and related constructs. For example, we might try to evaluate the convergent validity of a locally translated and modified version of the LibQUAL scale in the Persian language. Scores from the understudy version should correlate with scores of other questionnaires to the degree that one would expect. Assessing convergent validity is an iterative process: the more constructs are tested, the stronger the evidence towards the new LibQUAL being valid. Convergent validity is generally considered adequate if a correlation with an instrument measuring the same construct is >0.50.

The **discriminant validity** evaluates whether constructs that are not supposed to be related are unrelated. For example, we might try to evaluate the discriminant validity of the locally translated and modified version of the LibQUAL scale in the Persian language. Scores from the understudy construct (i.e., affect of service) should not correlate with scores of different and unrelated constructs (i.e., library as a place). A successful evaluation of discriminant validity shows that a construct is not highly correlated with other constructs designed to measure theoretically different concepts.

Fornell-Larcker Criterion is a rule suggested by Fornell and Larcker (1981) to assess convergent and discriminant validities. This criterion evaluates the distinctness between the latent variables in a model by a comparison of several statistical indicators.

Heterotrait–Monotrait Ratio (HTMT) is a statistical method suggested for evaluating discriminant validity.

Reliability

Reliability refers to the overall consistency of a test, questionnaire, or instrument. A questionnaire (i.e., LibQUAL) is considered to have high reliability if it produces similar results under consistent conditions. **Internal consistency** evaluates the consistency of results across items (questions) within a test (questionnaire). **Cronbach's alpha** is one of the most common measures used to evaluate internal consistency.

Composite reliability (also known as construct reliability) is a measure of internal consistency. It is like Cronbach's alpha and evaluates the overall consistency of items (questions) within a test (questionnaire). Cronbach's alpha is usually preferred while performing Exploratory factor analysis (EFA). However, composite reliability is usually preferred while performing confirmatory factor analysis (CFA).

Composite reliability is calculated by combining all the true score variances and covariances in the item variables (questions) related to constructs, and by dividing this sum by the total variance in the construct.

Factor analysis

Factor analysis is a statistical method used to describe the variability and modeling of observed variables and their statistical structure. According to factor analysis assumptions, there are several underlying factors that can explain the interrelationships among the observed variables in a model. Factor analysis is sometimes called “dimension reduction”. You can reduce the “dimensions” of your data into one or more “super-variables,” also known as unobserved variables or latent variables. These deeper concepts aren’t immediately obvious. They might represent traits or tendencies that are hard to measure, such as “service quality”.

Factor analysis isn’t a single technique, but a family of statistical methods that can be used to identify the latent factors driving observable variables. There are two types of factor analysis: exploratory and confirmatory factor analysis

Exploratory factor analysis (EFA)is a statistical method used to uncover the underlying structure of a relatively large set of items (variables). The overarching goal of EFA is to identify the underlying relationships between measured variables. It is commonly used by researchers when developing a scale (a questionnaire) and serves to identify a set of latent constructs (factors) underlying a range of measured variables. It should be used when the researcher has no a priori hypothesis about factors or patterns of measured variables. For example, you are developing a survey to evaluate the quality of services for a school library. You need to evaluate the primary observations by an EFA to uncover the latent variables and reduce the number of items to a more accurate model of variables.

Confirmatory factor analysis (CFA)is a statistical method used to verify the factor structure of a set of observed variables. CFA allows the researcher to test the hypothesis that a relationship between observed variables and their underlying latent constructs exists. the CFA serves to estimate the structure of an instrument (questionnaire), verifying how well the measured variables (questions) represent the number of constructs. Generally, the CFA is used when there is a previous study that tells us the dimensionality of that instrument. For example, we would have a European study that uses an EFA to verify the instrument’s dimensionality and you use a CFA to verify how well this structure happens with American data.

Factor loading is a numerical measure that describes how strongly a variable from the original research data is related to a given factor. Loadings are generally expected to be greater than 0.5.

Structural equation modeling (SEM)

SEM is a powerful, multivariate statistical technique used increasingly in scientific investigations to test and evaluate causal relationships. **SEM** has been widely used by librarians to test various hypotheses with multiple variables including public library services and use; satisfaction; quality of library services; acceptance of mobile library applications; and library success.

SEM refers to the construction of a model.In this model, different aspects of a phenomenon (i.e., service quality) are theorized to be related to one another with a structure (path). This structure is a

system of equations, but it is usually designed on paper or using a computer with arrows and symbols. The structure implies statistical and often causal relationships between variables, error terms and can include multiple equations. The equation (or equations) in SEM are mathematical and statistical properties that are implied by the model and its structural features and then estimated with statistical algorithms using experimental or observational data.

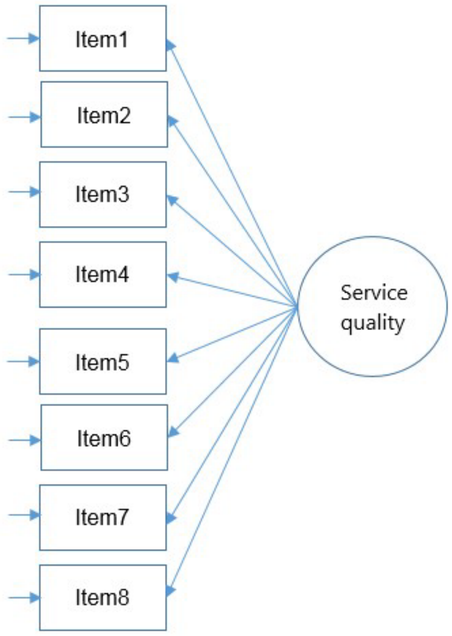

SEM usually involves two statistical methods: CFA and path analysis. **CFA** estimates the underlying factors and latent variables (i.e., satisfaction and attitude). **Path analysis** explores casual relationships among variables by creating path diagrams.

Partial least squares (PLS) is an efficient and optimal regression method to reduce the number of explanatory variables to a small set of predictors. In this study, we used PLS approach to analyze the initial pool of 45 candidate items to a valid and reliable scale with 23 items.

Average Variance Extracted (AVE) is commonly used to validate constructs. In statistics, AVE is a measure of the amount of variance that is captured by a construct in relation to the amount of variance due to measurement errors.

Path coefficient indicates the direct effect of a variable assumed to be a cause (for example library environment) on another variable assumed to be an effect (for example quality of services). Path coefficients are standardized because they are estimated from correlations. An arrow indicates the direction between case and effect in a model. From the image below, 1 is assumed as a cause for 2, 3 and 4 and 4 is an effect produced by direct or indirect effect of 1, 2, 3.

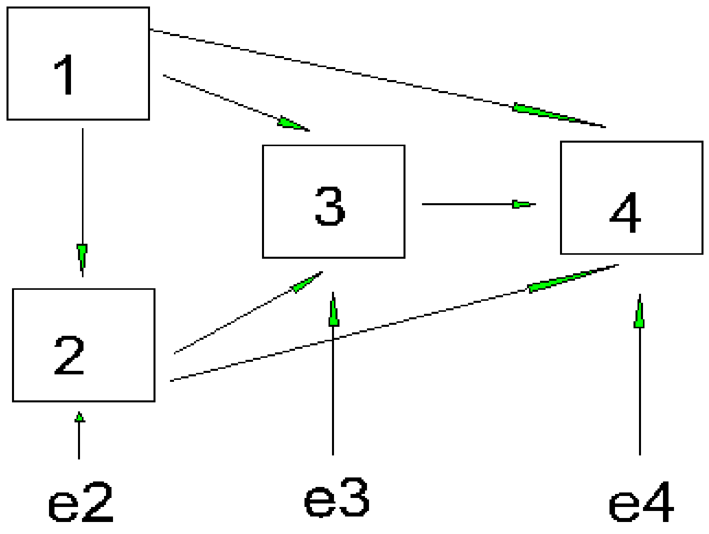

Bootstrapping creates subsamples with randomly drawn observations from the original set of data (with replacement). The subsample is then used to estimate the path analysis and PROCESS model. This procedure is repeated until a large number of random subsamples has been created, typically about 10,000.The parameter estimates obtained from the subsamples are used to derive the 95% confidence intervals for significance testing.
